# A Call to Improve Conditions for Conducting Holistic Review in Graduate Medical Education Recruitment

**DOI:** 10.15694/mep.2019.000076.1

**Published:** 2019-04-03

**Authors:** Christopher Williams, Brian Kwan, Anne Pereira, Ebony Moody, Steven Angus, Jehan El-Bayoumi

**Affiliations:** 1 George Washington University; 2 Department of Hospital Medicine; 3 University of Minnesota Medical School in Minneapolis; 4 United Healthcare Group; 5 University of Connecticut School of Medicine; 6 Rodham Institute

**Keywords:** residency recruitment, holistic review, graduate medical education, fellowship recruitment, application services, GME

## Abstract

This article was migrated. The article was marked as recommended.

The authors identify several components of the application for residency and fellowship recruitment that impede progress toward conducting holistic review in graduate medical education (GME). As well, important differences between undergraduate (UME) and graduate medical education (GME) recruitment are discussed. The authors call for inclusion of questions about family background and disadvantaged status in the GME application, which is a standard practice within applications for health professions. The second recommendation encourages a question about non-cognitive strengths. Many specialties have adopted standardized letters of recommendation (SLORs) or standardized letters of evaluation (SLOEs) that collect this information from letters writers. Programs and applicants would benefit from uniformity across specialties. The authors also call for a centralized, searchable database that provides applicants with each program’s mission, educational goals, and alumni outcomes. To support this paradigm shift in GME recruitment, reinforced with new Accreditation Council for Graduate Medical Education standards in July 2019, a task force should provide theoretical, evidence-based reasoning, along with development of new tools.

## Perspective

Conducting holistic review remains an ongoing challenge in graduate medical education (GME) recruitment due to many structural and cultural barriers ( Gliatto, Leitman, Muller, 2016). Defined as an individualized assessment of an applicant’s capabilities through balanced judgment of experiences, attributes, and academic metrics (EAM), holistic review has no shortage of benefits for individual applicants, learning environments, and programmatic educational aims seeking to fulfill accreditation standards ( Witzburg, Sondheimer, 2013; Mahon, Henderson, Kirch, 2013; Dore, Roberts, White, 2016). Yet, limitations of the residency and fellowship application, along with other factors related to organizational leadership and constraints can explain why a major shift toward holistic review has not occurred in GME, unlike in undergraduate medical education (UME). We will discuss how changes on three major fronts can improve the position of GME to assess attributes and experiences, while fulfilling mission-driven recruitment goals ( Table 1).

**Table 1.** Recommendations for Improve Conditions for Conducting Holistic Review in GME

1. Standardized questions about family background and disadvantaged status • Do you wish to be considered a disadvantaged applicant by any of your designated programs that may consider such factors (social, economic, or educational)? [yes or no] • What was the income level of your family during the majority of your time from birth to age 18? [choice of 14 income categories] [used to determine EO] • Were you required to contribute to the overall family income (as opposed to working primarily for your own discretionary spending?) [yes, no, or decline to answer] • Have members of your immediate family ever used U.S. federal or state assistance programs? [yes, no, don’t know, or decline to answer] • How have you paid, or did you pay for your postsecondary education? [percentages for academic scholarship, financial need-based scholarship, student loan, other loan, family contributions, applicant contribution, and other] • In what area did you spend the majority of your life from birth to age 18? • Do you believe that this area was medically underserved? • Were you approved for the AAMC Fee Assistance Program (FAP)? • Did you receive a Pell Grant? [yes, no] 2. Assessment of non-cognitive attributes 3. Centralized database of programs’ mission, educational aims, and alumni outcomes 4. National task force on holistic review

The Electronic Residency Application Service (ERAS) is the single major provider of application services for United States (US) GME residency and fellowship programs. Its application components have remained consistent over time: the USMLE or COMLEX transcript, the medical school transcript, the Medical Student Performance Evaluation (MSPE), letters of recommendation (LORs), personal statement, photo, and the MyERAS application (e.g. demographic data, research, educational history, previous training/employment). Together, these documents can facilitate holistic review. Race/ethnicity and gender are important metrics of diversity that, when used in combination with other individualized attributes, can enable programs to match a diverse cohort that enriches the learning environment and patient care delivery. Language skills, previous work experiences such as a former non-MD healthcare provider entering residency, advanced educational degrees, or geographic familiarity are some examples of how information available in the current application can prove valuable to programs. In addition, the personal statement and LORs are cited among highly important factors for interview selection ( National Residency Matching Service, 2019). However, the current MyERAS application lags other application services for health professions in the type of uniform data that it collects.

Questions about family background and disadvantaged status are standard for applications in other health fields: allopathic and osteopathic medical school, public health, pharmacy, nursing, podiatry, occupational therapy, optometry, physical therapy, and dentistry. In fact, most of these use the same application service - Liaison International - and benefit from a shared question bank ( Table 2) ( Schools of Public Health Application Service, 2019).

**Table 2.** Questions for Public Health Applicants Using Schools of Public Health Application Service (SOPHAS) in the Personal Background Information Section

1. Check if any of the following apply to you: • I graduated from a high school from which a low percentage of seniors received a high school diploma. • I graduated from a high school at which many of the enrolled students are eligible for free or reduced-price lunches. • I am from a family that receives public assistance (e.g. Aid to Families with Dependent Children, food stamps, Medicaid, public housing) or I receive public assistance. • I am from a family that lives in an area that is designated as a Health Professional Storage Area or a Medically Underserved Area. • I participated in an academic enrichment program funded in whole or in part by the Health Careers Opportunity Program. • I am a high-school drop-out who received AHS diploma or GED. • I am from a school district where 50% or less of graduates go to college or where college education is not encouraged. • I am the first generation in my family to attend college (neither my mother nor my father attended college). • I have a diagnosed physical or mental impairment that substantially limits my participated in educational experiences and opportunities offered by a college. • English is not my primary language. • I was accepted to the health professions program after academic reassessment at the completion of remedial courses. • Your parent’s family income falls within the table’s guidelines and you are considered to have met the criteria for economically disadvantaged. 2. To determine if you come from an economically disadvantaged background, you are asked to compare your parental family’s size of household (number of exemptions listed on parent’s Federal 1049 income tax forms) and adjusted gross income against the chart provided in the link below. The chart is based on 200 percent of federal low-income poverty guidelines. You should use your parent’s most recent tax forms regardless of age. Your parent’s family income falls within the table’s guidelines and you are considered to have met the criteria for economically disadvantaged. [yes, no] 3. What is the type of geographic area where you were raised? [urban, large city, mid-size city, large town, small town, isolated rural, do not wish to report]

Texas Medical & Dental Schools Application Service (TMDSAS) - the application service for all Texas public medical, dental, and veterinary schools - contains several sections on family and socioeconomic background, including first generation, college graduate status ( Texas Medical and Dental Schools Application Service, 2019). A service of the Association of American Medical College (AAMC), the American Medical College Application Service (AMCAS) uses valid measures of parental educational attainment and occupation to determine the Socioeconomic Status (SES) Education-Occupation (EO) Indicator for AMCAS applicants with the intended goal of “identify(ing) applicants who are the most socioeconomically disadvantaged.” (Association of American Medical Colleges, 2019; Grbic, Jones, Case, 2015). Applicants designated as EO-1 (applicants whose parents’ highest educational attainment is less than a bachelor’s degree/first-generation college graduate and whose background is from lowest SES group) and EO-2 (parental “service, clerical, skilled and unskilled” occupation, and at least a bachelor’s) are reported to medical schools. Information about applicants’ contribution to family income, family history of using US federal or state assistance, self-financing of education, and economically disadvantaged status would greatly expand the tools available for GME programs to conduct holistic review.

The Medical Student Performance Evaluation (MSPE) is technically considered a supporting document rather than part of the MyERAS application. Although intended to provide a summary of students’ personal attributes, experiences, and academic accomplishments, the MSPE does not fully lend itself to holistic review (Association of American Medical Colleges, 2019). Roughly one-half of residency program directors ostensibly use the MSPE to assess characteristics such as integrity, professionalism, intrinsic motivation, and reliability and dependability for interview decisions. However, program directors also expressed the lowest satisfaction with availability of tools to measure these characteristics (Association of American Medical Colleges, 2019). The Noteworthy Characteristics section is limited to no more than three bullets of two sentences or fewer discussing applicant attributes. Schools are instructed to comment upon professionalism, including any lapses. The MSPE is characterized by limited comparability due to non-uniformity across United States (USMS) and international (IMS) medical schools. The percentage of students in each medical school class that fall into defined performance categories (e.g. pass, high pass, and honors) vary greatly ( Osborn, Mattson , Yanuck, et al., 2016). Difficulty comparing information across medical schools was the greatest “pain point” for program directors (Association of American Medical Colleges, 2019).

Many specialties have chosen to adopt standardized letters of recommendation (SLORs) and standardized letters of evaluation (SLOEs) to capture more meaningful information such as fit for specialty and soft skills that can lead to individualized assessments. Otolaryngology began requiring residency applicants to provide a paragraph discussing their specific interest in each program but abandoned this requirement in 2018. Dermatology, emergency medicine, internal medicine, otolaryngology, plastic surgery, and orthopedic surgery have either SLORs or SLOEs. For example, the Emergency Medicine SLOE requests that letter writers comment on “relevant non-cognitive attributes such as leadership, compassion, positive attitude, professionalism, maturity, self-motivation, likelihood to go above and beyond, altruism, recognition of limits, conscientiousness, etc.” (Council of Residency Directors in Emergency Medicine, 2019). Internal medicine recommends program directors’ fellowship LORs detail applicant characteristics such as level of engagement and self-initiative, as well as skills sought beyond training requirements ( Alweis et al., 2017). The orthopedic SLOR contains percentile ranges for team-based skills, professionalism, initiative and drive, and commitment to specialty (The American Orthopaedic Association, 2019). Although research has shown positive effects because of standardization, challenges related to another component of the application - United States Medical Licensing Examination (USMLE) scores - impede major progress toward holistic review ( O’Connor et al., 2018; Kaffenberger et al., 2016).

The use of test scores is widespread for application screening. Sixty-four percent of residency programs across all specialties use United States Medical Licensing Examination (USMLE) Step 1 cutoff scores ( National Residency Matching Service, 2019). Except for four specialties (family medicine, plastic surgery, pathology, and thoracic Surgery), each has ranked USMLE Step 1/COMLEX Level 1 score as the most important factor (or tied with another factor) for considering applicants to interview ( Mcgaghie, Cohen, Wayne, 2011). Critics have opposed the use of USMLE altogether for residency recruitment based on validity (“neither structured, coherent, nor evidence based”), misappropriation of intended use (“designed to contribute to medical licensure decisions,” not residency recruitment), poor predictive power for residency performance, and potential to undermine “more holistic evaluation of the skills, attributes, and behaviors sought in future health care providers.” ( Mcgaghie, Cohen, Wayne, 2011; Wong, 2011; Bowe, Schmalbach, Laury, 2017; Gumbert, Guzman-Reyes, Pivalizza, 2016). Although assessing the validity of each of these claims is beyond the scope of this perspective, the use of USMLE for interview decisions is not a settled question. No overwhelming evidence warrants determinative use of USMLE in GME recruitment. We acknowledge that programs may use USMLE scores as a proxy for future Board performance to satisfy Accreditation Council for Graduate Medical Education (ACGME) standards for overall Board passage rates. Programs may also seek to manage an ever-increasing applicant pool (“application inflation”), which means that screening on USMLE score becomes the only practical or rational strategy for programs ( Pereira et al., 2016). Regardless of their influence, these factors should not figure into recruitment decisions as a matter of principle.

We propose two practical changes to the application based on trends that we discussed earlier. The first is including a section on family background and disadvantaged status, which is a step toward modernizing the GME application ( Table 1). We developed our recommendations based on revisions to family and socioeconomic background questions within the AMCAS application. Individuals with a history of social, economic, or educational challenges can be viewed in light of their resilience and academic accomplishments. Since the AAMC owns both AMCAS and ERAS, this is the most natural fit. Through matching of the AAMC identification number, responses in the original AMCAS application could be pre-populated, where available, for applicant review and modification for applicants’ MyERAS application. GME programs vary greatly in approaches to faculty and resident involvement in application review, interview, and ranking decisions with some allowing more liberal access to the entire application than others. Responses to these highly sensitive questions should be highly regulated by the program and their use limited to their stated purpose.

We also propose an expansion of the application to include a section on non-cognitive attributes for all specialties. The most practical approach involves selecting no more than three to five attributes that represent the applicant’s non-cognitive strengths from an extensive pre-populated list and allowing brief commentary on each attribute. Whether applicants, letter writers, or schools provide information on these attributes should be left up to the UME and GME communities to determine. There are advantages and disadvantages for each source. Although applicant self-assessment could be valuable in light of qualitative statements in the MSPE and LORs, programs may find it to be the most biased and ascribe little utility to it. On the other hand, the personal statement is highly regarded as an important factor for interview decisions ( Mcgaghie, Cohen, Wayne, 2011). Letter writers who presumably know the applicant well would be well-positioned to assess these attributes, but the completion of a separate form may involve duplicate work. Programs would likely welcome institutional assessment of applicants’ unique noncognitive skills. However, the response may not be able to capture deep personal insight.

Until now, we have discussed how changes to the application can facilitate holistic review. Equally critical is a change in recruitment priorities. As of July 1, 2019, a program “in partnership with its Sponsoring Institution, must engage in practices that focus on mission-driven, ongoing, systematic recruitment and retention of a diverse and inclusive workforce of residents, fellows (if present), faculty members, senior administrative staff members, and other relevant members of its academic community” ( Accreditation Council for Graduate Medical Education, 2019). This signals a major change on the part of ACGME to fundamentally reshape GME recruitment. Compared to current common program requirements in which “recruitment” never appears, the ACGME outlines expectations for a) “recruitment and retention of minorities underrepresented in medicine and medical leadership,” b) the program director’s role in recruitment and retention, and c) the Program Evaluation Committee’s assessment to recruit and retain a diverse workforce ( Accreditation Council for Graduate Medical Education, 2019). Program educational aims as currently described in ACGME standards should reflect a) programs’ distinguishing training features from others in the same specialty, b) the community needs, “with the ultimate goal of addressing these needs and health disparities” c) alumni outcomes, d) concurrence with institutional/departmental mission, and e) responsiveness to dynamic circumstances and ongoing assessment ( Accreditation Council for Graduate Medical Education, 2019; Accreditation Council for Graduate Medical Education, 2019). However, a major barrier threatens the success of this new paradigm shift - no tool exists that eases the way in which applicants can assess their fit with a program’s mission and educational goals.

Thus, we propose that applicants should have access to a centralized database allowing easy sorting and comparison of programs’ educational aims to identify optimal training environments to support career goals. Although many software (e.g. Fellowship and Residency Electronic Interactive Database, National Residency Matching Program (NRMP), ERAS, ACGME - Accreditation Data System, Doximity, TexasSTAR) house information about programs, varying from minimum USMLE requirements to artificial rankings, program aims do not appear as a feature on which applicants can sort. We propose that a medical education organization integrate such a tool within an existing service, while requiring programs to provide some data demonstrating achievement (e.g. alumni outcomes) of stated goals.

We also propose a national task force to explore program challenges and opportunities for holistic review in GME. Although medical schools have benefitted from tools for holistic review and the AAMC’s leadership in this space, UME and GME are markedly different. Besides the shortcoming in the GME application that we have previously discussed, the large size of each medical school class relative to the incoming class size for GME means that ensuring diversity of each GME program cohort is a more challenging proposition. Applicants are spread over many more programs than there are medical schools. GME does not have the same tools as UME at its disposal. There are no financial aid packages or scholarships in GME to attract applicants that will increase diversity. Salary is generally based on postgraduate year. Moreover, the “All-In” policy of the NRMP Main Residency Match - placing all or none of open positions in the Match - arguably constrains programs from increasing diversity through directed recruitment. Unlike in US medical schools in which international students make up one percent or less for matriculants for M.D.- (surmised using state of legal residence) and D.O.-granting schools, GME programs are more heavily reliant on international medical graduates (IMGs), defined as applicants who received medical school training abroad (Association of American Medical Colleges, 2019; American Association of Colleges of Osteopathic Medicine, 2019). IMGs constituted 43% of filled positions in internal medicine (categorical), 30% in family medicine, and 20% in pediatrics (categorical) for first year postgraduate year positions (PGY-1) ( National Residency Matching Service, 2019). Non-U.S. citizen IMGs filled 14% of the filled PGY1 positions in the NRMP Main Residency Match. Thus, one charge of this task force should be to articulate how holistic review best works in this milieu and to make recommendations for addressing structural barriers.

Additionally, this task force should be charged with a) providing evidence-based and theoretical reasoning for benefits of holistic review to individual learners, learning environments, and public health. and b) identifying forms of explicit and implicit bias that have led to current challenges in recruitment. For example, USMLE cutoffs likely unintentionally screen out those from lower socioeconomic classes and family income since standardized tests tend to favor examinees from high-income families (The Chronicle of Higher Education, 2019; College Board, 2019). Confronting biases requires meaningful introspection and the courage to lead discussions beyond medical education to those about structural inequity. Black or African American and Hispanic or Latino medical students’ percentage of U.S. medical school matriculants has hardly changed since 1980; respectively, 6.0% versus 7.1% and 4.9% versus 6.3% (Association of American Medical Colleges, 2019). These groups have not largely benefitted from the immense diversification of medical school student bodies. Despite this, Black physicians, who are racially-concordant with patients have been associated with a 19% reduction in the black-white health disparity in cardiovascular mortality (The National Bureau of Economic Research, 2019). Other charges should be to c) identify research questions that require further study, d) disseminate or create innovative tools, including appropriate use of family and disadvantage responses, and e) provide the conditions for ongoing dialogue and resource support, including support for institutions whose states currently ban consideration of race/ethnicity. Even in states without such bans, some institutions and programs choose to remove photos and race/ethnicity fields during the application review process.Finally, this task force should be closely aligned with ACGME accreditation standards and the focus areas of the Clinical Learning Environment Review (CLER) program. Although we would hope that all programs would be invested in furthering holistic review, the reality is that some programs may feel disconnected from this discussion for assorted reasons: residing in a relatively homogenous region, less competitive programs, small size, limited resources, or program culture. The proposed tools have the potential to enrich applicant files regardless of whether programs fully buy-in. Hence, if recruitment of a diverse workforce in skills, attributes, and experiences can tie to domains of patient safety and health care quality, then proponents are more likely to have the ear of critics. In its 2018 National Report of CLER Findings, the ACGME states that, “CLEs face significant challenges in implementing change at the speed and magnitude needed to keep pace with, or ideally anticipate, the future of health care delivery” ( Accreditation Council for Graduate Medical Education, 2019). To move forward, new tools and paradigms will be needed to catalyze rapid change in GME recruitment and training. Broadly-defined, diversity is our greatest strength as a nation and profession that, once availed, best positions us to meet the current and future needs of patients and communities.

## Take Home Messages

Several practical solutions can address challenges for conducting holistic review in GME: questions about family background and disadvantage status and non-cognitive strengths. A task force should seek to further improve conditions.

## Notes On Contributors

Chris Williams, MPH is a public health consultant with the Rodham Institute, School of Medicine and Health Sciences, George Washington University, Washington, District of Columbia. ORCID: https://orcid.org/0000-0001-5767-8048

Brian Kwan, MD is Health Sciences Associate Professor at Department of Hospital Medicine, University of California and Staff Physician/Hospitalist, VA San Diego Healthcare System in San Diego, California.

Anne Pereira, MD, MPH is Assistant Dean for Curriculum and Associate Professor of Medicine at the University of Minnesota Medical School in Minneapolis, Minnesota. ORCID: https://orcid.org/0000-0003-4203-6311

Ebony Moody is Senior Clinical Administrative Coordinator, United Healthcare Group, Clinical Operations, Boston, MA.

Steven Angus, MD is Professor, Department of Medicine, and Designated Institutional Official at University of Connecticut School of Medicine, Farmington, Conn.

Jehan El-Bayoumi, MD is Professor of Medicine and Founding Director of the Rodham Institute, School of Medicine and Health Sciences, George Washington University, Washington, District of Columbia.

## References

[ref1] Accreditation Council for Graduate Medical Education (2019). Common Program Requirements. Available at: https://www.acgme.org/What-We-Do/Accreditation/Common-Program-Requirements( Accessed February 6, 2019).

[ref2] Accreditation Council for Graduate Medical Education (2018). National Report of Findings 2018: Executive Summary. https://www.acgme.org/Portals/0/PDFs/CLER/CLER_2018_Executive_Summary_DIGITAL_081418.pdf( Accessed February 7, 2019).

[ref3] Accreditation Council for Graduate Medical Education (2019). The ACGME Program Self-Study: Developing Your Program Aims. https://www.acgme.org/Portals/0/PDFs/SelfStudy/SSAimsIPLK.pdf( Accessed February 6, 2019).

[ref4] Alweis R. Collichio F. Milne C.K. (2017) Guidelines for a Standardized Fellowship Letter of Recommendation. The American Journal of Medicine. 30(5), pp.606–611. 10.1016/j.amjmed.2017.01.017 28189466

[ref5] American Association of Colleges of Osteopathic Medicine . https://www.aacom.org/docs/default-source/data-and-trends/2017-aacomas-applicant-matriculant-profile-summary-report.pdf?sfvrsn=4f072597_12 2017 AACOMAS Applicant and Matriculant Profile Summary Report.( Accessed February 6, 2019).

[ref6] Association of American Medical Colleges . Effective Practices for Using the AAMC Socioeconomic Status Indicators in Medical School Admissions. https://www.aamc.org/download/330166/data/seseffectivepractices.pdf( Accessed February 6, 2019).

[ref7] Association of American Medical Colleges . Matriculants to U.S. Medical Schools by State of Legal Residence, 2009-2010 through 2018-2019. https://www.aamc.org/download/321462/data/factstablea4.pdf( Accessed February 6, 2019).

[ref8] Association of American Medical Colleges . Medical Student Performance Evaluation (MSPE). https://www.aamc.org/members/gsa/54686/gsa_mspeguide.html( Accessed February 6, 2019).

[ref9] Association of American Medical Colleges . Results of the 2016 Program Directors Survey Current Practices in Residency Selection. https://members.aamc.org/eweb/upload/Program%20Directors%20Survey%20Report.pdf( Updated 2016. Accessed February 6, 2019).

[ref10] Association of American Medical Colleges . Trends in Racial and Ethnic Minority Applicants and Matriculants to U.S. Medical Schools, (1980-2016). https://www.aamc.org/download/484966/data/november2017trendsinracialandethnicminorityapplicantsandmatricu.pdf( Updated November 2017. Accessed February 7, 2019).

[ref11] Bowe S.N. Schmalbach C.E. Laury A.M. (2017) The State of the Otolaryngology Match: A Review of Applicant Trends, “Impossible” Qualifications, and Implications. Otolaryngology-Head and Neck Surgery. 156(6), pp.985–990. 10.1177/0194599817695804 28319452

[ref12] College Board . (2013). College-Bound Seniors: Total Group Profile Group. http://media.collegeboard.com/digitalServices/pdf/research/2013/TotalGroup-2013.pdf( Accessed February 7, 2019).

[ref13] Council of Residency Directors in Emergency Medicine . The Standardized Letter of Evaluation (SLOE). https://www.cordem.org/esloe( Accessed February 6, 2019).

[ref14] Dore K.L. Roberts C. Wright S. (2016) Widening perspectives: reframing the way we research selection. Advances in Health Sciences Education. 22(2), pp.565–572. 10.1007/s10459-016-9730-5 27815761

[ref15] Gliatto P. Leitman I.M. Muller D. (2016) Scylla and Charybdis. Academic Medicine. 91(11), pp.1498–1500. 10.1097/ACM.0000000000001247 27254007

[ref16] Grbic D. Jones D.J. Case S.T. (2015) The Role of Socioeconomic Status in Medical School Admissions. Academic Medicine. 90(7), pp.953–960. 10.1097/ACM.0000000000000653 25629949

[ref17] Gumbert S.D. Guzman-Reyes S. Pivalizza E.G. (2016) More About the Role of USMLE Step 1 Scores in Resident Selection. Academic Medicine. 91(11), pp.1469. 10.1097/ACM.0000000000001401 27779522

[ref18] Kaffenberger J.A. Mosser J. Lee G. (2016) A Retrospective Analysis Comparing the New Standardized Letter of Recommendation in Dermatology with the Classic Narrative Letter of Recommendation. Journal of Clinical and Aesthetic Dermatology. 9(9), pp.36–42. PMC5110327 27878060

[ref19] Mahon K.E. Henderson M.K. Kirch D.G. (2013) Selecting Tomorrow’s Physicians. Academic Medicine. 88(12), pp.1806–1811. 10.1097/ACM.0000000000000023 24128626

[ref20] Mcgaghie W.C. Cohen E.R. Wayne D.B. (2011) Are United States Medical Licensing Exam Step 1 and 2 Scores Valid Measures for Postgraduate Medical Residency Selection Decisions. Academic Medicine. 86(1), pp.48–52. 10.1097/ACM.0b013e3181ffacdb 21099388

[ref21] National Residency Matching Service . Main Residency Match Data and Reports. http://www.nrmp.org/main-residency-match-data( Accessed February 6, 2019).

[ref22] O’Connor A. Williams C. Dalal B. (2018) Internal Medicine Fellowship Directors’ Perspectives on the Quality and Utility of Letters Conforming to Residency Program Director Letter of Recommendation Guidelines. Journal of Community Hospital Internal Medicine Perspectives. 8(4), pp.173–176. https://doi.org/10.1080%2F20009666.2018.1500424 30181820 10.1080/20009666.2018.1500424 PMC6116145

[ref23] Osborn M.B. Mattson J. Yanuck J. (2016) Ranking Practice Variability in the Medical Student Performance Evaluation. Academic Medicine. 91(11), pp.1540–1545. 10.1097/ACM.0000000000001180 27075499 PMC5937982

[ref24] Pereira A.G. Chelminski P.R. Chheda S.G. (2016) Application Inflation for Internal Medicine Applicants in the Match: Drivers, Consequences, and Potential Solutions. The American Journal of Medicine. 129(8): pp.885–891. 10.1016/j.amjmed.2016.04.001 27154785

[ref25] Schools of Public Health Application Service . (2019). SOPHAS Applicant Help Center. https://help.liaisonedu.com/SOPHAS_Applicant_Help_Center( Accessed March 6, 2019).

[ref26] Texas Medical and Dental Schools Application Service . (2019). Application Handbook. https://www.tmdsas.com/Forms/ApplicationHandbookEY2019.pdf( Accessed February 6, 2019).

[ref27] The American Orthopaedic Association . Standardized Letter of Recommendation (SLOR). https://www.aoassn.org/aoaimis/AOANEW/Residents/Standardized_Letter_of_Recommendation/AOANEW/Residents/Standardized_Letter_of_Recommendation_Form.aspx?hkey=e6cad4dc-6002-4885-b9a8-152854b5a674

[ref28] The Chronicle of Higher Education . Standardized Tests Favor Students from High-Income Families. https://www.chronicle.com/blogs/letters/standardized-tests-favor-students-from-high-income-families/( Updated June 27, 2018. Accessed February 2, 2019).

[ref29] The National Bureau of Economic Research . Does Diversity Matter for Health? Experimental Evidence from Oakland. www.nber.org/papers/w24787.pdf Updated September 2018. ( Accessed February 7, 2019).

[ref30] Witzburg R.A. Sondheimer H.M. (2013) Holistic Review - Shaping the Medical Profession One Applicant at a Time. New England Journal of Medicine. 368(17), pp -1565–1567. 10.1056/NEJMp1300411 23574032

[ref31] Wong J.G. (2011) The Role of USMLE Scores in Selecting Residents. Academic Medicine. 86(7), pp.793–794. 10.1097/ACM.0b013e31821d3df3 21715986

