# Supplementary material for: A Call to Improve Conditions for Conducting Holistic Review in Graduate Medical Education Recruitment
Source: MedEdPublish (2016). 2019 Apr 3;8:76. [Version 1] doi: 10.15694/mep.2019.000076.1 (PMC10712609; doi:10.15694/mep.2019.000076.1)
Supplement: Peer review history

# Reviewer report: Reviewer response for version 1

**DOI:** 10.21956/mep.19811.r29830

**Published:** 2019-07-12

**Authors:** Trevor Gibbs

**Affiliations:** 1 AMEE

This review has been migrated. The reviewer awarded 4 stars out of 5 We ask many questions about undergraduate education and have been blamed for leaving postgraduate education out in the cold- hence I looked forward to reading this paper. It is a strong paper that explores or dares to inquire about aspects of GME no usually sort. I felt it gave a very passionate plea for the described holistic inquiry and would recommend it to all involved in GME curricula design and application.

Reviewer Expertise:

NA

No decision status is available

# Reviewer report: Reviewer response for version 1

**DOI:** 10.21956/mep.19811.r29829

**Published:** 2019-04-18

**Authors:** Samar Aboulsoud

**Affiliations:** 1 CAIRO UNIVERSITY, SCHOOL OF MEDICINE

This review has been migrated. The reviewer awarded 4 stars out of 5 Thank you for this interesting article. The authors boldly explored challenges in GME in a comprehensive and evidence based approach. I agree it's time to think of a change and do things differently. Although exploring the background of candidates in the way described by the authors can be seen as a double-sided weapon, I believe this can be instrumental in the recruitment process if a tool is carefully developed, implemented and monitored. I hope this article leads to more work not only in the US but also in other that will eventually help improve the physician training worldwide.

Reviewer Expertise:

NA

No decision status is available

# Reviewer report: Reviewer response for version 1

**DOI:** 10.21956/mep.19811.r29828

**Published:** 2019-04-11

**Authors:** Felix Silwimba

**Affiliations:** 1 University of Lusaka

This review has been migrated. The reviewer awarded 5 stars out of 5 Although this paper touched on the US graduate medical education admissions to make them more inclusive. i find the arguments applicable to my situation , in an African country. in this country representation in medical education can be biased based on ethnicity, urban/rural residence , type of prior education, disability and gender representation. it is a must article to be read by all medical educators concerned with equity.

Reviewer Expertise:

NA

No decision status is available
